# Supplementary material for: Knowledge, Attitudes, and Practices on COVID-19 Vaccination among General Adult Population in Malawi: A Countrywide Cross-Sectional Survey
Source: Vaccines (Basel). 2024 Feb 20;12(3):221. doi: 10.3390/vaccines12030221 (PMC10975966; doi:10.3390/vaccines12030221)
Supplement: Supplementary file 1 [file vaccines-12-00221-s001.zip › vaccines-2831142-supplementary.pdf]

vaccines

MDPI

Supplementary Documents

Supplementary 1, **Table S1**: COVID-19 disease burden and sample size
Source: Malawi COVID-19 epidemiological report, Epi Week 8 2022: NSO, 2020

District	Total COVID-19 confirmed cases	Population	Burden %	Sample unit/district
MACHINGA	1011	735438	0.14	54
THYOLO	1058	721456	0.15	58
NTCHEU	999	659608	0.15	58
DEDZA	1278	830512	0.15	60
CHIKWAWA	901	564684	0.16	63
NSANJE	528	299168	0.18	70
PHALOMBE	800	429450	0.19	
BALAKA	973	438379	0.22	
MWANZA	291	130949	0.22	
DOWA	1769	772569	0.23	
MULANJE	1646	684107	0.24	
MANGOCHI	2770	1148611	0.24	
KASUNGU	2418	842953	0.29	
CHIRADZULU	1072	356875	0.30	
CHITIPA	715	234927	0.30	120
NKHOTAKOTA	1373	393077	0.35	
NKHATABAY	1098	284681	0.39	152
SALIMA	1899	478346	0.40	157
MCHINJI	2395	602305	0.40	
KARONGA	1535	365028	0.42	
RUMPHI	1023	229161	0.45	
NTCHISI	1480	317069	0.47	184
ZOMBA	4008	746724	0.54	212
LILONGWE	19757	2,626,902	0.75	296
NENO	1043	138291	0.75	296
LIKOMA	118	14527	0.81	320
MZIMBA		940184	0.81	320
BLANTYRE	23332	1,251,484	1.86	735

Supplementary 2, **Table S2**: Sample sheet of Enumeration areas (EA) and households per district

	Rural		Urban		Sample/Rural		Sample/Urban	
DISTRICT	EAs	Households	EAs	Households	EAs	Households	EAs	Households
BLANTYRE CITY			24	384			15	480
BLANTYRE	24	384			8	256		
LILONGWE CITY			36	576			11	176
LILONGWE	36	576			8	128		
MZUZU CITY			24	384			14	224
ZOMBA	24	384			6	96		
ZOMBA CITY			24	384			8	128
MZIMBA	24	384			6	96		
SALIMA	21	336	3	48	4	64	3	96
NTCHISI	23	368	1	16	8	128	1	64
DEDZA	23	368	1	16	2	32	1	32
NKHATABAY	23	384	1	16	6	96	1	64
THYOLO	23	384	1	16	2	32	1	32
NENO	24	384	0	0	19	304		
MACHINGA	23	368	1	16	2	32	1	32
NTCHEU	23	368	1	16	2	32	1	32

CHIKWAWA	23	368	1	16	2	32	1	32
CHITIPA	22	352	2	32	4	64	2	64
NSANJE	22	352	2	32	2	32	2	40
LIKOMA	23	384	1	16	20	320		
